# Prevalence of Carpal Tunnel Syndrome During Pregnancy in Iran

**DOI:** 10.1016/j.jhsg.2026.101025

**Published:** 2026-04-30

**Authors:** Hesam Alitaleshi, Mojtaba Baroutkoub, Hossein Safaei, Mosayeb Soleymani

**Affiliations:** ∗ Department of Orthopedic Surgery, Shariati Hospital, Tehran University of Medical Sciences, Tehran, Iran; † Department of Orthopedic and Trauma Surgery, Shahid Beheshti University of Medical Sciences, Tehran, Iran; ‡ Department of Sports and Exercise Medicine, Rasool Akram General Hospital, Iran University of Medical Sciences, Tehran, Iran

**Keywords:** Carpal tunnel syndrome, EMG-NCV, Median nerve compression, Pregnancy, Prevalence

## Abstract

**Purpose:**

Carpal tunnel syndrome (CTS) is a common musculoskeletal disorder during pregnancy, second only to back pain. Although the prevalence in the general population is approximately 4%, a prevalence of up to 43% has been reported in pregnant women. This review aimed to assess and summarize reported point-prevalence estimates of CTS among pregnant women in Iran.

**Methods:**

Five observational studies conducted in Iran were included. Diagnostic approaches varied across studies. Clinical diagnosis was defined as the presence of characteristic symptoms (paresthesia, numbness, nocturnal symptoms in the median nerve distribution) with or without positive provocative tests (Tinel and/or Phalen). Electrodiagnostic confirmation required abnormal nerve conduction velocity and/or EMG findings consistent with median nerve compression at the wrist. Data were extracted for the entire sample, including trimester, parity, bilateral involvement, and diagnostic tools.

**Results:**

A total of 2065 pregnant women were assessed across the included studies. Based on clinical criteria, the estimated prevalence of CTS during pregnancy in Iran was approximately 10.5%. Subjective symptoms were reported in 10.84%, objective signs in 10.31%. Electrodiagnostic tests (EMG and nerve conduction velocity) confirmed CTS in 5.67% of these 2065 pregnant women. Bilateral involvement was observed in 47.7% of symptomatic patients. The highest prevalence was observed in the third trimester (64.4%), and most affected women were experiencing their first pregnancy (55%).

**Conclusions:**

Reported prevalence of CTS during pregnancy in Iranian studies varies substantially depending on diagnostic criteria and study design. Although this variability does not indicate systematic underdiagnosis or inconsistent reporting, it highlights methodological heterogeneity across studies. Importantly, there is a paucity of high-quality evidence addressing optimal treatment strategies for CTS during and after pregnancy, underscoring the need for well-designed clinical studies in this area.

**Type of study/level of evidence:**

Prognostic III

## Graphical abstract

**Figure:**
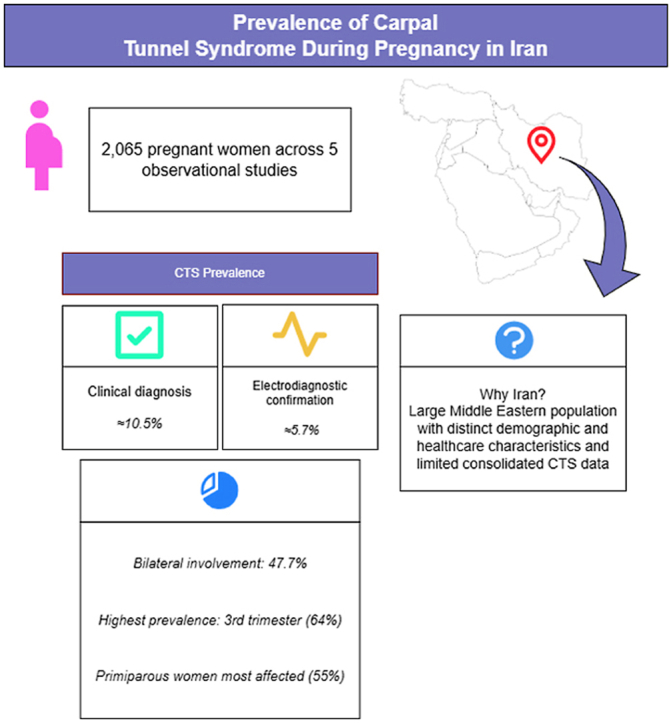

## Introduction

Pregnancy is a physiological process that may, nonetheless, be accompanied by musculoskeletal disorders. The most common musculoskeletal manifestations during pregnancy include low back pain, wrist pain, hip pain, and limb paresthesia. Several factors contribute to these symptoms, including weight gain, hormonal changes, fluid retention, postural alterations, and biomechanical changes.^1^

Carpal tunnel syndrome (CTS) is a common musculoskeletal problem during pregnancy, ranking second only to back pain.^2^ Although the prevalence of CTS in the general population is approximately 4%, studies have reported a pregnancy-related prevalence ranging from 7% to 43%.^3^ The condition is often bilateral and significantly more common during the third trimester.^4^

Iran represents a large and demographically distinct population within the Middle East, with substantial ethnic diversity and varying access to prenatal health care across regions. Understanding the epidemiology of pregnancy-related CTS in this setting contributes to global comparisons of diagnostic practices, population-specific risk patterns, and health system variability. Synthesizing national data allows assessment of regional variability in reported prevalence and diagnostic practices, which may differ from those observed in Western and other Middle Eastern populations. Therefore, the present study aimed to summarize and describe the reported prevalence of pregnancy-related CTS among pregnant women in Iran.

## Materials and Methods

### Study design

This study is a review aimed at investigating the prevalence of CTS during pregnancy in Iran. It is important to note that all included studies were predominantly cross-sectional; therefore, the reported estimates represent point or period prevalence of CTS during pregnancy rather than incidence of new-onset disease. Although pregnancy is a transient physiological state, prevalence remains an appropriate epidemiologic measure for describing the proportion of women affected at a given time point or within a specific trimester.

The design and implementation of the review followed the Preferred Reporting Items for Reviews and Meta-Analyses (PRISMA) 2020 guidelines to ensure transparency and reporting quality. Given the local scope and focus on domestic data, the review was not registered in international review registries such as PROSPERO.

### Eligibility criteria

Studies were eligible if they were observational in design (cross-sectional, cohort, or case-control), conducted in Iran, involved pregnant women, and reported original prevalence data for CTS. Publications in English or Persian were included. The exclusion criteria were as follows: (1) studies conducted in non-Iranian populations; (2) studies including nonpregnant participants; (3) review articles, case reports, editorials, or interventional studies; (4) studies lacking original prevalence data; (5) studies with insufficient diagnostic clarity for CTS; and (6) duplicate publications or overlapping data sets.

### Search strategy

A comprehensive and targeted search was conducted in international databases, including PubMed, Scopus, Web of Science, and Embase. To cover Persian-language publications, national databases such as SID.ir, Magiran, IranMedex, and ISC were also searched. The keywords used included: “Carpal Tunnel Syndrome,” “Pregnancy,” “Prevalence,” “CTS in pregnancy,” “Median nerve compression,” “Compression neuropathy,” “Iran,” and “Incidence.” However, studies reporting incidence were excluded unless prevalence data could be extracted. These terms were combined using Boolean operators (AND, OR) to broaden the search and identify studies on the prevalence of CTS among pregnant women in Iran. In Persian databases, equivalent terms were used. The search was conducted without time restrictions, and studies published up to September 2025 were included.

### Study screening

Study selection followed a 2-stage screening process in accordance with Preferred Reporting Items for Systematic Reviews and Meta-Analyses (PRISMA) guidelines. After removal of duplicates, titles and abstracts were independently screened by 2 reviewers. Records that clearly failed to meet the eligibility criteria were excluded. Full texts of potentially relevant articles were then assessed for eligibility. Disagreements were resolved through discussion or consultation with a third reviewer. Reasons for exclusion at the full-text stage are summarized in the PRISMA flow diagram (Fig.). The most common reasons for exclusion at the full-text stage were absence of prevalence data, inclusion of nonpregnant populations, and insufficient diagnostic definition of CTS.

**Figure:**
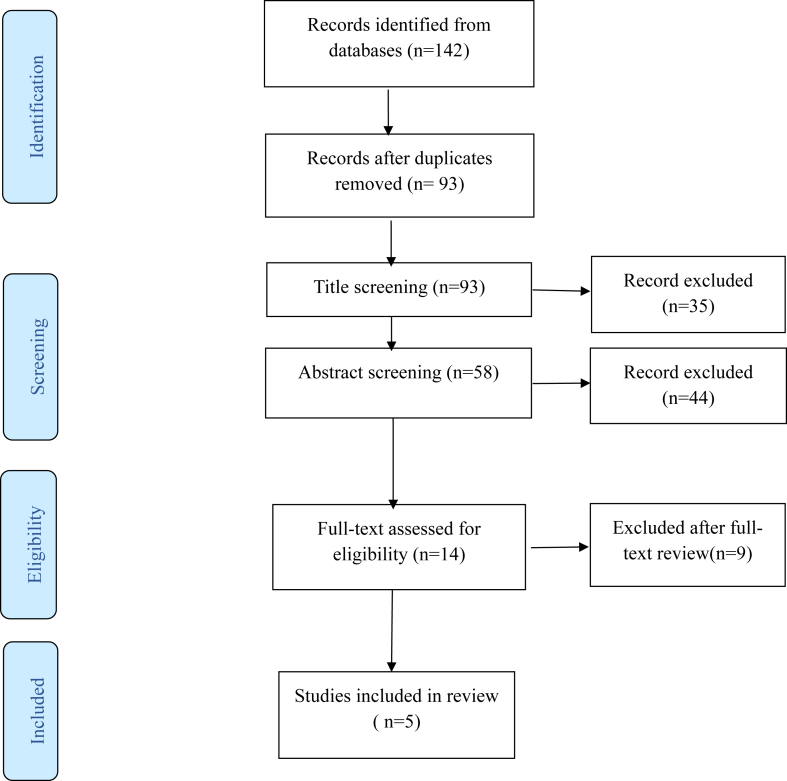
Preferred Reporting Items for Systematic Reviews and Meta-Analyses (PRISMA) flow diagram illustrating the study selection process.

### Data extraction

A structured data extraction form was developed to collect the following information: author name, year of publication, study type, sample size, CTS prevalence, pregnancy trimester, affected side (right or left), bilateral involvement, number of pregnancies, and diagnostic tool used to diagnose CTS. Data extraction was performed independently by 2 reviewers, and disagreements were resolved through discussion or referral to a third reviewer.

### Definition of diagnostic categories

For this review, CTS diagnoses were categorized into 2 predefined groups:

1. Clinical CTS: Defined as the presence of characteristic median nerve distribution symptoms (paresthesia, numbness, nocturnal symptoms affecting digits 1–3 ± radial half of the 4th digit) with at least 1 positive provocative test (Tinel and/or Phalen), when explicitly reported by the original study authors.
2. Electrodiagnostically confirmed CTS: Defined as abnormal median nerve conduction findings on nerve conduction velocity (NCV) testing and/or EMG, consistent with median nerve compression at the wrist, according to the criteria specified in each study (eg, prolonged distal motor latency, prolonged sensory latency, or abnormal median–ulnar latency comparison). Although there were minor variations in electrodiagnostic thresholds across studies, all electrodiagnostic diagnoses were based on objective median nerve conduction abnormalities at the wrist.

When studies reported subjective symptoms and objective signs separately, these were extracted independently and classified accordingly. No reclassification or reinterpretation of diagnostic criteria was performed beyond the definitions provided in the original studies.

### Data analysis

Extracted data included author, publication year, study type, sample size, CTS prevalence, pregnancy trimester, affected side, bilateral involvement, number of pregnancies, and diagnostic method. Descriptive analysis was performed. Where possible, CTS prevalence was stratified by pregnancy trimester, affected side, and number of pregnancies. Prevalence rates were reported as percentages, and the range of variation across studies was examined. Studies were also categorized based on diagnostic method (clinical vs electrodiagnostic) and study design. Due to heterogeneity in study design, diagnostic tools, and reporting formats, meta-analysis was not conducted, and the results remained descriptive.

## Results

### Overview of included studies

This review included 5 observational studies conducted in Iran that investigated the prevalence of CTS during pregnancy. Diagnostic approaches differed across studies. One study established the diagnosis based solely on clinical evaluation, defined by characteristic symptoms and physical examination findings. The remaining studies used a 2-step approach, in which clinically suspected cases were confirmed with electrodiagnostic testing, including NCV studies and/or EMG. In these studies, prevalence estimates were ultimately based on electrodiagnostically confirmed cases rather than clinical findings alone.

### Sample size and prevalence

Across the 5 studies, a total of 2065 pregnant women were assessed. The reported prevalence rates varied widely across studies, reflecting differences in diagnostic criteria, study design, and population characteristics. Detailed study-level results are presented in the Table.^5^, ^6^, ^7^, ^8^, ^9^ Overall prevalence estimates were calculated as pooled proportions (total number of cases divided by the total sample size across studies), rather than as averages of individual study percentages.

**Table:** Summary of Studies on the Prevalence of CTS During Pregnancy in Iran∗

First authors, year	Location (city)	Sample size	Diagnostic method/criteria	CTS symptoms (%)	Prevalence by EMG-NCV (%)
Meibody etal^7^ 2009	Isfahan	267	Clinical: symptoms and positive provocation tests.	19.5	-
Yazdanpanah et al 2012^5^	Yasuj	1508	Two-step: clinical screening, then EMG/NCV confirmation.	3.4	3.4
Khosrawi et al 2012^6^	Isfahan	100	Two-step: clinical, then EMG/NCV confirmation.	40	19
Shaafi et al 2006^8^	Tabriz	90	Two-step: clinical, then EMG/NCV confirmation.	50	16.7
Bahrami et al 2005^9^	Tehran	100	Two-step: clinical, then EDX confirmation.	36	17
Overall		2065		10.84	5.67

EDX, electrodiagnostic.

∗ Overall values represent pooled proportions calculated from aggregated numerators and denominators across studies and do not represent meta-analytic weighted means.

The largest included study (*n* = 1508) accounted for approximately 73% of the total pooled sample. Because 1 large study contributed most participants, the reported aggregate prevalence should be interpreted cautiously. Therefore, results are presented descriptively to avoid disproportionate statistical weighting and overestimation of precision.

### Bilateral involvement

Four studies reported data on bilateral CTS involvement. Among symptomatic patients, 47.7% exhibited bilateral signs and symptoms.

### Trimester-specific prevalence

Three studies stratified CTS prevalence by pregnancy trimester. Among 118 pregnant women diagnosed with CTS, the highest prevalence was observed in the third trimester (64.4%), followed by the second trimester (27.12%) and the first trimester (8.47%). These findings indicate that CTS symptoms are more frequently reported in later stages of pregnancy.

### Parity and CTS

Two studies examined the association between parity and CTS prevalence. Among affected women, 55% were experiencing their first pregnancy, 31% were in their second pregnancy, and 13% were in their third or higher-order pregnancy. This distribution may suggest increased susceptibility to CTS among primiparous women, although further studies are needed to validate this trend.

## Discussion

One of the challenging aspects of CTS is its diagnosis. However, electrodiagnostic testing is frequently used as a confirmatory tool; clinical evaluation remains the primary diagnostic standard in routine practice.^10^

As Graham^11^ noted in his study, in most patients diagnosed with CTS based solely on clinical history and physical examination, electrodiagnostic testing (such as nerve conduction studies) does not change the diagnostic likelihood.

Based on clinical criteria, the estimated prevalence of CTS during pregnancy in Iran is approximately 10%. However, considering that some Iranian studies reported subjective and objective findings separately, the prevalence should be interpreted within a range, from 10.84% based on subjective symptoms to 10.31% based on objective clinical signs.

The reported prevalence of pregnancy-related CTS varies considerably across the international literature, ranging from 2% to 62%. This wide variation is largely attributable to differences in study design, sample size, and, in particular, the diagnostic criteria employed.^12^, ^13^, ^14^, ^15^, ^16^, ^17^, ^18^, ^19^, ^20^ For instance, the highest prevalence (62%) was reported in a smaller study by Padua et al^12^ (*n* = 76) that used clinical diagnosis, a finding that warrants cautious interpretation given the limited sample size. In contrast, studies with larger cohorts generally report more moderate figures. Oliveira et al^13^ found that 23% of 482 pregnant women presented with signs and symptoms consistent with CTS, whereas Meems et al^14^ reported a symptom-based prevalence of 34% in 639 Dutch women. Similarly, Khan et al^15^ documented a prevalence of 30% in their study of 200 pregnant women.

Interestingly, some larger studies report lower estimates that align more closely with the pooled analysis of the present review. Smitha^16^ in India observed a 9% prevalence among 371 participants, and Allawi^17^ in Iraq reported a clinical prevalence of 10.8% in a cohort of 500 women. At the lower end of the spectrum, Finsen and Zeitlmann^18^ in Norway reported a prevalence of only 2%. However, their methodology—dividing the number of clinical cases by the total number of births during the study period—suggests this figure more accurately reflects incidence than point prevalence. The convergence of estimates from larger studies toward a more consistent range likely reflects reduced random error, underscoring the value of robust sample sizes in providing reliable epidemiological data.

A critical and consistent theme across the literature is the discrepancy between prevalence based on clinical signs and symptoms versus that confirmed by electrodiagnostic testing. Clinical criteria consistently yield higher estimates than neurophysiological confirmation. For example, Iranian studies report an EMG-NCV confirmed prevalence of 5.67%, which is approximately half of the clinically diagnosed cases. This pattern is echoed by Tupković et al,^19^ who found that while 30% (12/40) of women were clinically diagnosed, 75% of those cases (9 patients) were subsequently confirmed via neurophysiological testing. This diagnostic gap is further illustrated by the systematic review by Padua et al,^20^ which reported a clinical prevalence range of 31% to 62%, compared with a broader, notably lower electrodiagnostic range of 7% to 43%. These findings highlight the high sensitivity but potential lack of specificity in clinical diagnosis alone, suggesting that symptom-based estimates may overburden the true prevalence of pathophysiologically confirmed CTS. At the same time, neurophysiological testing may offer a more specific, albeit potentially less sensitive, benchmark.

An important consideration is whether all patients with electrodiagnostic findings indicative of median nerve involvement actually present with clinical symptoms. In a study by Afaq involving 75 pregnant women, only 6% of those who met electrodiagnostic criteria for CTS exhibited concurrent positive clinical signs.^21^ Conversely, Baumann et al^22^ demonstrated that although median nerve conduction was consistently prolonged in all pregnant participants compared with nonpregnant controls, only 11% met the strict electrodiagnostic criteria for CTS; notably, half of those individuals were symptomatic at the time of assessment or developed symptoms later. These findings underscore the nuanced relationship between subclinical nerve compression and symptomatic disease, suggesting that physiological changes of pregnancy may affect nerve function without necessarily crossing the threshold for clinical diagnosis.

Regarding the timing of onset, most studies report a higher prevalence of CTS in the third trimester. This pattern was evident in the present review, where 64.4% of 118 Iranian participants experienced symptoms in the third trimester. Supporting this, Yaseen et al^23^ in Pakistan reported a third-trimester prevalence of 38.8% compared with 28.9% in the first, and Mateen et al^24^ found that 55% of affected patients were in their third trimester. However, this temporal distribution is not universal; Wright et al’s^25^ study paradoxically reported higher prevalence during the first and second trimesters, suggesting that factors such as fluid retention patterns or study population characteristics may influence the timing of symptom onset.

Bilateral involvement emerges as a consistently prominent feature of pregnancy-related CTS. In the current review, 47.7% of cases demonstrated bilateral symptoms. This finding aligns with a substantial body of literature reporting even higher rates: Rozali et al^26^ documented 63% bilateral involvement, whereas Meems et al and Turgut et al^27^ each reported 70%, and Mondelli et al^28^ found the highest rate at 78%. Slightly lower but comparable estimates were provided by Oliveira et al (40%) and Padua et al (45%), reinforcing that bilateral presentation is a characteristic hallmark of CTS in pregnancy.

Several methodological limitations across the literature warrant consideration. Iranian studies, in particular, have not consistently employed standardized diagnostic tools such as validated questionnaires. Instruments such as the Boston Carpal Tunnel Questionnaire, commonly used to assess treatment response, and the CTS-6, a practical diagnostic aid requiring no specialized training, remain underutilized.^29^ Furthermore, although ultrasonography has emerged as a valuable diagnostic modality for CTS with established validity,^30^^,^^31^ it has not been incorporated into Iranian prevalence studies of pregnancy-related CTS. Another notable gap is the limited evaluation of gestational weight gain as a contributing factor. Among Iranian studies, only Meibody et al^7^ assessed this variable by comparing it with a control group,^25^^,^^32^^,^^33^ despite evidence implicating weight gain in CTS pathophysiology.

Overall, Iranian data indicate a moderate prevalence of pregnancy-related CTS that is comparable to similarly sized international studies. Nevertheless, methodological heterogeneity, especially in diagnostic thresholds and electrodiagnostic confirmation, substantially influences reported rates. These findings emphasize the need for standardized diagnostic frameworks incorporating clinical, electrophysiological, and sonographic criteria, as well as larger, methodologically robust studies to clarify CTS epidemiology during pregnancy.

## Conclusion

The reported prevalence of CTS during pregnancy in Iranian studies ranges between approximately 5% (electrodiagnostic confirmation) and 10% (clinical diagnosis). Variability is primarily attributable to differences in diagnostic criteria and study methodology rather than systematic underdiagnosis. The current evidence base is limited by methodological heterogeneity and by the concentration of data in a single large study. Future research should prioritize standardized diagnostic definitions and evaluation of treatment strategies during and after pregnancy.

### Limitations

A substantial proportion of the pooled sample (73%) originated from a single large study. Consequently, the aggregated prevalence estimate may be disproportionately influenced by this study’s methodology and population characteristics. Therefore, the overall prevalence should be interpreted with caution.

Furthermore, no pooled meta-analytic prevalence estimate was calculated, because 1 large study accounted for the majority of participants.

## Conflict of interest

No benefits in any form have been received or will be received related directly to this article.
